# *Bacillus* Responses to Plant-Associated Fungal and Bacterial Communities

**DOI:** 10.3389/fmicb.2020.01350

**Published:** 2020-06-23

**Authors:** Sofija Andrić, Thibault Meyer, Marc Ongena

**Affiliations:** Microbial Processes and Interactions Laboratory, Terra Teaching and Research Center, Gembloux Agro-Bio Tech, University of Liège, Gembloux, Belgium

**Keywords:** *Bacillus*, rhizosphere, bioactive secondary metabolites, microbial interaction, biocontrol, molecular cross-talk, phenotype modulation

## Abstract

Some members of root-associated *Bacillus* species have been developed as biocontrol agents due to their contribution to plant protection by directly interfering with the growth of pathogens or by stimulating systemic resistance in their host. As rhizosphere-dwelling bacteria, these bacilli are surrounded and constantly interacting with other microbes via different types of communications. With this review, we provide an updated vision of the molecular and phenotypic responses of *Bacillus* upon sensing other rhizosphere microorganisms and/or their metabolites. We illustrate how *Bacillus* spp. may react by modulating the production of secondary metabolites, such as cyclic lipopeptides or polyketides. On the other hand, some developmental processes, such as biofilm formation, motility, and sporulation may also be modified upon interaction, reflecting the adaptation of *Bacillus* multicellular communities to microbial competitors for preserving their ecological persistence. This review also points out the limited data available and a global lack of knowledge indicating that more research is needed in order to, not only better understand the ecology of bacilli in their natural soil niche, but also to better assess and improve their promising biocontrol potential.

## Introduction

Some *Bacillus* species of the *B. subtilis* complex are plant-associated and important members of the microbiome (Mendes et al., 2013; Müller et al., 2016; Fierer, 2017). During the last decades, their potential use as biocontrol agents with protective activity toward economically important plant pathogens has been highlighted thereby representing a promising alternative to chemical pesticides (Expósito et al., 2017; Fan et al., 2017; Finkel et al., 2017; Fira et al., 2018; Köhl et al., 2019). The efficacy of such bacilli in plant protection, as well as their constant presence in the strongly competitive rhizosphere niche, are due to their high potential to synthesize a wide range of volatile organic compounds (VOCs) and soluble bioactive secondary metabolites (BSMs). High structural diversity is observed in the patterns of VOCs formed by *Bacillus* (Caulier et al., 2019; Kai, 2020) but also in BSMs which can be either ribosomally synthesized and post-translationally modified like bacteriocins and lantibiotics or enzymatically formed via multi-modular mega-enzymes as in the case of polyketides (PKs), di-peptides or cyclic lipopeptides (CLPs) (Harwood et al., 2018; Kaspar et al., 2019; Rabbee et al., 2019). A prime role of some soluble BSMs and volatiles in plant protection is related to their strong antimicrobial activity leading to direct antagonism against plant pathogens (Raaijmakers and Mazzola, 2012; Borriss, 2015; Chowdhury et al., 2015a; Fan et al., 2018; Caulier et al., 2019; Rabbee et al., 2019; Kai, 2020). A second important biocontrol-related trait of those compounds is their ability to trigger an immune reaction in the host plants which leads to systemic resistance (Induced SR) rendering the plant less susceptible to pathogen infection (Pieterse et al., 2014; Chowdhury et al., 2015a; Fan et al., 2018; Caulier et al., 2019; Rabbee et al., 2019). An additional role of BSMs is also linked to an efficient plant root colonization ability of *Bacillus* which indirectly protects the plant by decreasing the space and nutrient availability for pathogens (Raaijmakers et al., 2010; Borriss, 2015; Nayak et al., 2020). Some BSMs also contribute to colonization since they are involved in the developmental processes of *Bacillus* social motility and biofilm formation (Raaijmakers and Mazzola, 2012; Borriss, 2015; Pandin et al., 2017).

As rhizosphere-dwelling bacteria, these plant-associated bacilli are influenced by various environmental factors, such as temperature, pH, moisture, light, and nutrient composition dictated by plant exudation (Santoyo et al., 2017). In this competitive niche, *Bacillus* species are also surrounded by and constantly interacting with a myriad of other (micro)organisms (Mendes et al., 2013; Traxler and Kolter, 2015; Fierer, 2017; Schmidt et al., 2019). In this review, we illustrate the diversity of BSMs produced by different *Bacillus* species and how this metabolome and phenotypic traits dictating ecological fitness can be impacted upon interaction with other fungal and bacterial microorganisms. The outcomes of volatile-based microbial interactions, in general, have been recently reviewed (Schmidt et al., 2015; Tyc et al., 2017). However, when dealing with interactions involving bacilli, information is scarce concerning possible changes in VOCs production upon cross-talk or perception of volatiles produced by other microorganisms (Chen et al., 2015; Tahir et al., 2017; Martínez-Cámara et al., 2019). Thus, we focus hereafter on interactions based on cross-talks mediated by the perception of soluble metabolites.

## Diversity and Bioactivities of *Bacillus* BSMs

In the comparative genomic era, numerous adjustments have been done in the last years to clarify the phylogeny of the *B. subtilis* complex, which includes, among others, species, such as *B. velezensis*, *B. amyloliquefaciens*, *B. atrophaeus*, *B. subtilis* subspecies *subtilis*, *B. licheniformis*, *B. pumilus*, and *B. siamensis* with potential as biocontrol agents (Expósito et al., 2017; Fira et al., 2018; Maksimov et al., 2020), and which led to some confusion in species names but also to misassignments (Dunlap et al., 2016; Fan et al., 2017; Harwood et al., 2018; Du, 2019; Torres Manno et al., 2019). Many isolates, such as strains FZB42, QST713, or SQR9 formerly assigned to the *B. subtilis* or *B. amyloliquefaciens* species have been re-classified as *B. velezensis* representing the model species for plant-associated bacilli (Dunlap et al., 2016; Fan et al., 2017). A large part of the genome of these species is devoted to the production of antimicrobial compounds with up to 12% annotated as involved in the synthesis of bioactive secondary metabolites (Molinatto et al., 2016; Fan et al., 2017; Pandin et al., 2018).

**FIGURE 1:**
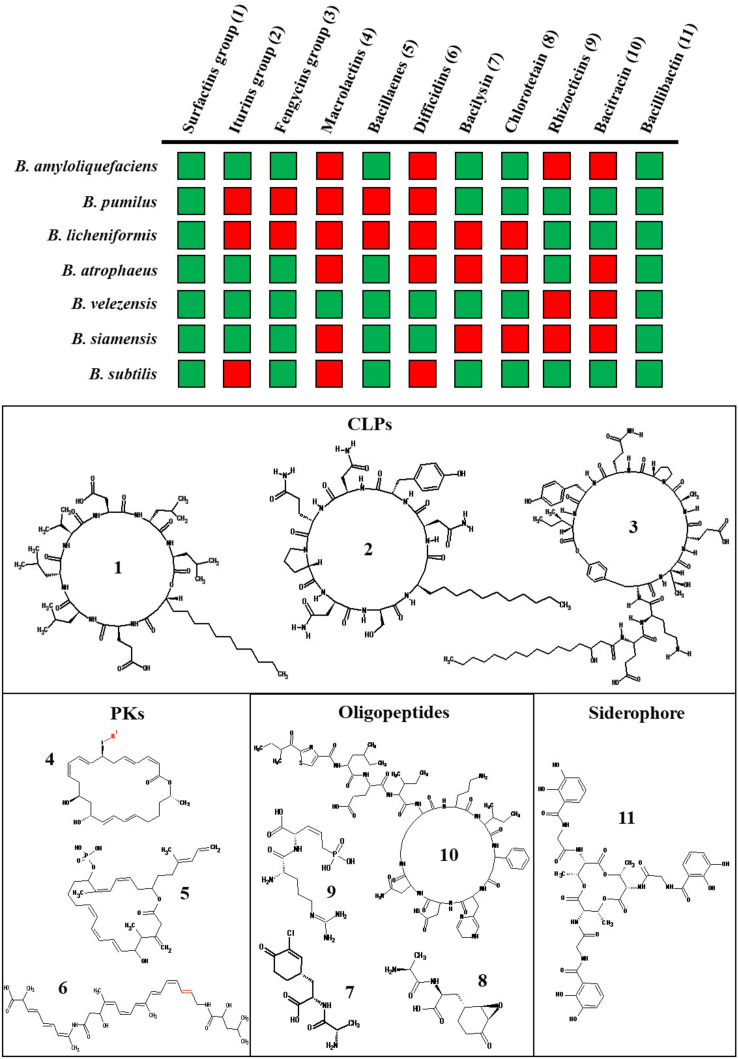
Main non-ribosomal BSMs produced by the various species in the *B. subtilis* complex. The BSMs production is indicated for the following species *B. subtilis, B. siamensis, B. velezensis, B. atrophaeus, B. amyloliquefaciens, B. pumilus*, and *B. licheniformis* by a green square whereas red square indicates an absence of production of the BSMs in this species (Zhao and Kuipers, 2016; Fan et al., 2017; Harwood et al., 2018; Du, 2019; Torres Manno et al., 2019). The surfactins, iturins, and fengycins groups include lichenycin (1;AA1:L-Gln) and pumilacidin (1;AA4: L-Leu, AA7 = I-Ile); mycosubtilin (2;AA6: D-Ser, AA7 = L-Thr) and bacillomycin (2; AA6: D-Ser, AA7 = L-Asn); maltacin (4;AA1: L-Ser), agrastatin (4;AA10: L-Val) and plipastatin (4;AA9: D-Tyr), respectively. The structure of the representative metabolite is indicated by a number and represented below. The possible variations in the PKs structure are highlighted in red. For the macrolactin family, the main variants are R = H; CO-CH_2_-COOH; CO-CH_2_-CH_2_-COOH or 6-*O*-succinyl-β-glucose (for review see Piel, 2010).

Non-ribosomal metabolites are synthesized either by polyketide synthases (PKS) or non-ribosomal peptide synthase (NRPS), both acting as assembly lines catalyzing different steps for the incorporation of amino acid residues (Dutta et al., 2014; Winn et al., 2016; Bozhüyük et al., 2019). The three main families of *Bacillus* CLPs are surfactins, fengycins, and iturins (Figure 1). According to this limited number of families identified so far, the structural diversity of *Bacillus* CLPs may appear quite limited compared to other bacterial genera, such as *Pseudomonas*, for which many more different groups have been discovered (Geudens and Martins, 2018; Götze and Stallforth, 2020). However, reduced specificity of adenylation domains allows substitutions at specific places in the peptide chain and the NRPS machinery can bind different fatty acids with various chain lengths in the initiation step leading to co-production of various homologs within the three families as illustrated in Figure 1 (Kraas et al., 2010; Bozhüyük et al., 2019). Interestingly, some CLP peptidic variants are synthesized through species-specific clusters, like pumilacidin and lichenysin which are only produced respectively by *B. pumilus* and *B. licheniformis* (Figure 1).

The three different types of CLPs retain specific but complementary functions considering biocontrol efficiency and, more generally, ecological fitness of the producing strains. By contributing to motility and biofilm formation, surfactins are involved in colonization of plant tissues which indirectly allow *Bacillus* to outcompete phytopathogens for space and nutrients. Surfactins are also involved in the molecular cross-talk with the host and it is well-characterized as an elicitor of plant immunity leading to ISR (Ongena and Jacques, 2008; Henry et al., 2011; García-Gutiérrez et al., 2013; Cawoy et al., 2015; Chowdhury et al., 2015a). Direct antibiotic activity of surfactins at biologically relevant concentrations toward soil-dwelling or plant-associated microbes has been only occasionally reported (Qi et al., 2010; Liu et al., 2014). By contrast, fengycins and iturins are best characterized for their antifungal activities against a wide range of plant pathogens (Caulier et al., 2019; Rabbee et al., 2019). This is mainly due to their ability to perturb fungal cell membrane integrity resulting in cytoplasm leakage and finally hyphae death and inhibition of spore germination (Chitarra et al., 2003; Romero et al., 2007; Deleu et al., 2008; Etchegaray et al., 2008; Gong et al., 2015; Gao et al., 2017; Zhang and Sun, 2018). The three CLPs retain some selectivity but may also act synergistically to inhibit fungal growth (Liu et al., 2014). The lipid composition of the plasma membrane could explain differences in the sensitivity of fungal targets to one or more CLPs (Wise et al., 2014; Fiedler and Heerklotz, 2015).

Besides lipopeptides, most species of the *B. subtilis* group also produce other non-ribosomal oligopeptide derivatives, such as bacilysin, chlorotetaine, bacitracins, and rhizocticins which are known to be efficient as antibacterial compounds targeting cell wall biosynthesis (Zhao and Kuipers, 2016). The siderophore bacillibactin is highly conserved in the *B. subtilis* group (Figure 1) and is induced in response to iron limitation in the environment. It allows *Bacillus* to efficiently acquire Fe^3+^ and other metals (Miethke et al., 2006, 2008; Li et al., 2014) thereby depriving phytopathogens of this essential element (Miethke et al., 2006; Niehus et al., 2017).

Polyketide biosynthesis is performed by successive condensation of small carboxylic acids mediated by core domains of the corresponding enzyme machinery but some PKs are synthesized via hybrid NRPS/PKS systems leading to the integration of amino acid residues (Piel, 2010; Olishevska et al., 2019). The three main PKs produced by *Bacillus* are difficidins, macrolactins, and bacillaenes, the latter being more widespread across species (Figure 1). The main PKs role is related to their antibacterial activity via the ability to inhibit protein biosynthesis in numerous phytopathogenic bacteria but certain antifungal activity has been reported for bacillaenes and macrolactins (Caulier et al., 2019; Olishevska et al., 2019).

Ribosomally synthetized BSMs encompass bacteriocins and lantibiotics including plantazolicin, subtilin, ericin, mersacidin, amylolysin, and amylocyclicin that are specifically produced by some species or strains (Brötz et al., 1998; van Kuijk et al., 2012; Arguelles Arias et al., 2013; Scholz et al., 2014; Torres Manno et al., 2019). These BSMs are responsible for growth inhibition of Gram-positive bacteria by acting via different modes of action (Abriouel et al., 2011; Acedo et al., 2018).

## Perception of Fungi Triggers the Production of Appropriate BSMs

Several works have illustrated the impact of phytopathogenic fungi on BSMs production by soil bacilli. Some *B. amyloliquefaciens*, *B. velezensis*, and *B. subtilis* strains respond to the presence of antagonistic fungi by stimulating the production of the antifungal CLPs fengycins and/or iturins (Table 1). Not only the production of specific CLPs varies in a species-dependent manner but it is also highly dependent on the interacting fungal species. For example, much higher production of iturins and fengycins by *B. subtilis* 98S was observed in confrontation with *Pythium aphanidermatum* and *Fusarium oxysporum* but not with *Botrytis cinerea* (Cawoy et al., 2015). Further, upon interaction with fungi, some *B. velezensis* strains (SQR9, FZB42, and S499) overproduced either iturins or fencycins (Li et al., 2014; Chowdhury et al., 2015b; Kulimushi et al., 2017). For instance, Li et al. (2014) showed that when confronted with *Sclerotinia sclerotiorum, B. velezensis* SQR9 overproduces bacillomycin D (iturin family), but not fengycins. An overproduction of bacillomycin along with a reduced production of fengycins was also reported by Chowdhury et al. (2015b) upon *B. velezensis* FZB42 interaction with *Rhizoctonia solani* in the rhizosphere of lettuce plants. Differentially, Kulimushi et al. (2017), showed that strains S499 and FZB42 improved production of fengycin but not iturins upon interaction with *Rhizomucor variabilis*. Most of these studies also indicated that fengycins and iturins are the main BSMs responsible for antifungal activities (Table 1). Thus, *Bacillus* cells could specifically sense the presence of fungal competitors and accordingly overproduce appropriate antifungal BSMs to outcompete the interacting fungi. Moreover, besides modulating the production of fengycins and iturins, some strains of *B. velezensis* (SQR9, FZB42, and QST713) and *B. subtilis* (B9-5) may overproduce surfactins when sensing phytopathogenic fungi (Li et al., 2014; Chowdhury et al., 2015b; DeFilippi et al., 2018; Pandin et al., 2019). In support to this hypothesis, surfactin production of *B. velezensis* FZB42 was highly induced in the presence of fungal pathogen *R. solani* in the lettuce rhizosphere where it was found as the main produced compound (Chowdhury et al., 2015b). A similar response was recorded when *B. velezensis* SQR9 was confronted with *S. sclerotiorum* and *Phytophthora parasitica* (Li et al., 2014) or when *B. subtilis* B9-5 interacted in liquid medium with *Rhizopus stolonifer* (DeFilippi et al., 2018). In contrast to fengycins and iturins, surfactins are not strong direct antifungal metabolites in biologically relevant concentrations (Raaijmakers and Mazzola, 2012). Thus, it stays unclear why *Bacillus* responded by surfactin overproduction to the presence of antagonistic fungi. A possible explanation could be rooted in its global role promoting the rhizosphere and thereby, contributing to competition for nutrients and space with the interacting fungi (Ongena and Jacques, 2008; Rabbee et al., 2019).

**TABLE 1:** Change in expression and bioactivity of BSMs produced by members of *B. subtilis* group, upon interaction with fungal species.

**BSMs**	**Change in expression**	**Involvement in antifungal activity**	***Bacillus* species (strains)**	**Fungal species**	**References**
Fengycins	0	Yes	*B. subtilis* (98S)	*B. cinerea*	Cawoy et al., 2015
	+	Yes	*B. subtilis* (98S)	*F. oxysporum*	Cawoy et al., 2015
	+	No	*B. subtilis* (98S)	*P. aphanidermatum*	Cawoy et al., 2015
	+	Yes	*B. velezensis* (S499)	*R. variabilis*	Kulimushi et al., 2017
	+	Yes	*B. velezensis* (FZB42)	*R. variabilis*	Kulimushi et al., 2017
	0	Yes	*B. velezensis* (QST713)	*R. variabilis*	Kulimushi et al., 2017
	+	Yes	*B. velezensis* (SQR9)	*Verticillium dahliae*	Li et al., 2014
	+	Yes	*B. velezensis* (SQR9)	*F. oxysporum*	Li et al., 2014
	+	Yes	*B. velezensis* (SQR9)	*Phytophthora parasitica* var. *nicotianae*	Li et al., 2014
	-	Mediating the plant defense expression	*B. velezensis* (FZB42)	*R. solani*	Chowdhury et al., 2015b
	+	ND	*B. subtilis* (B9-5)	*R. stolonifer*	DeFilippi et al., 2018
	+	ND	*B. subtilis* (B9-5)	*Fusarium sambucinum*	DeFilippi et al., 2018
	+	ND	*B. subtilis* (B9-5)	*V. dahliae*	DeFilippi et al., 2018
	+	ND	*B. velezensis* (QST713)	*Trichoderma aggressivum f. europaeum*	Pandin et al., 2019
Iturins	0	Yes	*B. subtilis* (98S)	*B. cinerea*	Cawoy et al., 2015
	+	Yes	*B. subtilis* (98S)	*F. oxysporum*	Cawoy et al., 2015
	+	No	*B. subtilis* (98S)	*P. aphanidermatum*	Cawoy et al., 2015
	+	No	*B. velezensis* (SQR9)	*V. dahliae*	Li et al., 2014
	+	No	*B. velezensis* (SQR9)	*S. sclerotiorum*	Li et al., 2014
	+	Yes	*B. velezensis* (SQR9)	*F. oxysporum*	Li et al., 2014
	+	Yes	*B. velezensis* (SQR9)	*P. parasitica*	Li et al., 2014
	+	Mediating the plant defense expression	*B. velezensis* (FZB42)	*R. solani*	Chowdhury et al., 2015b
Surfactins	+	Yes	*B. velezensis* (SQR9)	*S. sclerotiorum*	Li et al., 2014
	+	Yes	*B. velezensis* (SQR9)	*R. solani*	Li et al., 2014
	+	Yes	*B. velezensis* (SQR9)	*Fusarium solani*	Li et al., 2014
	+	Yes	*B. velezensis* (SQR9)	*P. parasitica*	Li et al., 2014
	+	Mediating the plant defense expression	*B. velezensis* (FZB42)	*R. solani*	Chowdhury et al., 2015b
	+	ND	*B. subtilis* (B9-5)	*R. solani*	DeFilippi et al., 2018
	+	ND	*B. subtilis* (B9-5)	*F. sambucinum*	DeFilippi et al., 2018
	+	ND	*B. subtilis* (B9-5)	*V. dahliae*	DeFilippi et al., 2018
	+	ND	*B. velezensis* (QST713)	*T. aggressivum f. europaeum*	Pandin et al., 2019
Bacillibactin	+	Yes	*B. velezensis* (SQR9)	*V. dahliae*	Li et al., 2014
	+	No	*B. velezensis* (SQR9)	*S. sclerotiorum*	Li et al., 2014
	+	No	*B. velezensis* (SQR9)	*F. oxysporum*	Li et al., 2014
	+	Yes	*B. velezensis* (SQR9)	*R. solani*	Li et al., 2014
	+	Yes	*B. velezensis* (SQR9)	*F. solani*	Li et al., 2014
	+	Yes	*B. velezensis* (SQR9)	*P. parasitica*	Li et al., 2014

*“0” indicates no changes, “+” enhanced and, “–”decreased BSMs production by Bacillus upon interaction with fungi. “Yes” indicates fungitoxic activity, “No” no antifungal activity, “ND” indicates that BSMs with antifungal activity are not detected.*

Even though the siderophore bacillibactin is produced by all members of the *B. subtilis* species complex (Figure 1), its possible overproduction upon microbial interactions has been poorly investigated. Interestingly, the work of Li et al. (2014) showed that *B. velezensis* SQR9 overproduces bacillibactin when grown in presence of a range of fungi including *V. dahliae*, *S. sclerotiorum*, *F. oxysporum*, *R. solani*, *F. solani*, and *P. parasitica*. This may be interpreted as a response of the bacterium to some iron-limitation in the medium caused by the fungi via the release of their own chelatants.

In *B. subtilis*, the expression of many BSMs biosynthesis genes is transcriptionally fine-tuned by compound-specific regulation but also by global regulators governing the transition to crucial developmental processes like motility, biofilm formation and sporulation (Inaoka et al., 2009; López et al., 2009; Vargas-Bautista et al., 2014). Fungal triggers may affect both types of regulatory systems involved in BSMs production. For instance, upon sensing *F. verticillioides*, the global stress-related regulator SigB is activated in *B. subtilis* NCIB3610 which in return enhances surfactin production (Bartolini et al., 2019). In interaction with *F. culmorum* under biofilm-conducive conditions, *B. subtilis* Bs12 down-regulates the expression of the *sin*R gene known as a repressor of biofilm formation which also negatively regulates surfactin production (Kearns et al., 2005; Khezri et al., 2016; Zhi et al., 2017). These observations strongly suggest that specific soluble signals, emitted by fungal pathogens, could be perceived by bacilli which in turn modulate BSMs synthesis. As observed by Bartolini et al. (2019), cells of the *Bacillus* colony, physically close to the fungal culture, responded to signals by over-expressing genes coding for transcription factors involved in CLPs synthesis regulation. In contrast, colony cells positioned on the opposite side of the fungi did not react to the fungus (Bartolini et al., 2019). This phenomenon indicates that the specific fungal metabolite diffuses on a short distance and has an influence on closely located *Bacillus* cells. Currently, no fungal compounds have been identified as triggers of BSM stimulation in *Bacillus*. Nonetheless, few commonly produced metabolites by *Fusarium* species were suggested to modify *Bacillus* behavior. It was shown that two cyclic depsipeptides (enniatins B1 and enniatins A1) and a pyrone (lateropyrone) had an antagonistic effect on *B. subtilis* growth (Ola et al., 2013). Fusaric acid also modified antibacterial activity of *B. mojavensis* but it was not related to a decrease in the production of specific BSMs (Bacon et al., 2004, 2006; Bani et al., 2014). These metabolites could also play a triggering role at sub-inhibitory concentration and could have an inducible effect on the range of *Bacillus* responses as has been shown for other signal metabolites (Bleich et al., 2015; Liu et al., 2018).

## *Bacillus* Phenotype Is Modulated Upon Perception of Bacterial Competitors

Some BSMs may also act as molecular determinants driving outcomes of interactions between *B. subtilis* and bacterial competitors as illustrated for the bacillaene polyketide displaying an essential protective role for survival in competition with *Streptomyces* soil isolates (Straight et al., 2007; Barger et al., 2012). However, there are few direct evidences for enhanced expression of BSMs upon interbacteria interactions. The only convincing examples involve the interaction of plant-associated bacilli with plant pathogens, such as *Ralstonia solanacearum* (Almoneafy et al., 2014) and *Pseudomonas fuscovaginae* (Kakar et al., 2014). In these two studies, improved expression of surfactin, bacilysin, and iturin biosynthesis genes were observed when *Bacillus* and pathogens were grown together in dual-cultures. Nevertheless, no clear indication about the enhanced production of the aforementioned BSMs based on their quantification nor improved antibacterial activities of *Bacillus* was presented as a result of this interaction.

Interestingly, at the phenotypical level, the development of soil bacilli is differentially altered upon sensing other bacteria from the same natural environment. Some of these phenotypical changes can be associated or due to a modulated production of specific BSMs. First, exogenous antibiotics or signals may stimulate biofilm formation which depends, to some extent, on surfactin production (López et al., 2009) and which may be viewed as a defensive response against exogenous toxic compounds and/or infiltration by competitors (Flemming et al., 2016; Townsley and Shank, 2017; Molina-Santiago et al., 2019). For instance, *B. subtilis* increased its relative subpopulation of biofilm matrix-producing cells in response to small molecules secreted by other bacterial species (López et al., 2009; Shank et al., 2011). The same phenomenon was illustrated for thiazolyl peptides emitted by closely related species, such as *B. cereus* and putatively formed by other soil microbes, such as *Streptomyces* isolates (Bleich et al., 2015). However, no change in surfactin production associated with the stimulation of biofilm was reported in these studies.

Besides biofilm formation, other mechanisms drive bacteria to initiate protective responses upon the detection of competitors. The flagellum-independent sliding motility is considered as an adaptive mechanism that allows bacterial cells to physically relocate in the context of a competitive interaction (Wadhams and Armitage, 2004; Jones et al., 2017; McCully et al., 2019). Upon sensing *S. venezuelae*, the *B. subtilis* ability to slide was increased (Liu et al., 2018). It depends in part on the production of surfactin (Grau et al., 2015; van Gestel et al., 2015) but a potential boost in lipopeptide synthesis upon the perception of the *Streptomyces* challenger was not demonstrated. Chloramphenicol and derivatives produced by *S. venezuelae* were identified as molecular triggers acting at subinhibitory concentrations for inducing *Bacillus* motility (Liu et al., 2018).

Multiple bacteria promote sporulation in *B. subtilis* which represents another example of alteration of the physiological development of this species. In a context of distant interactions, exogenous siderophores accelerate the differentiation of *Bacillus* cells into spores. It was notably shown for enterobactin from *E. coli* and for ferrioxamine E produced by *Streptomycetes* (Grandchamp et al., 2017). In iron-limited environments, *B. subtilis* cells would thus respond by taking up those “piratable” siderophores and start sporulating. This is not a general response to xenosiderophores since for instance, pyochelin from *Pseudomonas* does not affect *Bacillus* sporulation (Molina-Santiago et al., 2019). Nevertheless, the ability of siderophores to alter cellular differentiation in *B. subtilis* suggests that those molecules are likely to mediate complex microbial interactions in iron-depleted conditions, as often met in a soil environment. However, induction of *B. subtilis* sporulation by other bacteria may also occur in a cell-to-cell contact situation. Upon interaction with *P. chlororaphis*, its type VI secretion system acted as a trigger for sporulation, independently from its established role as cargo for delivering toxic effectors into the target *Bacillus* cells (García-Bayona and Comstock, 2018; Molina-Santiago et al., 2019).

That said, interspecies interactions may also result in inhibition rather than in stimulation of key developmental processes determining the fate of *Bacillus* multicellular communities. As an example, 2,4-diacetylphloroglucinol, a broad-spectrum antibiotic synthesized by fluorescent *Pseudomonas*, alters colony morphology, inhibits biofilm formation and sporulation in *B. subtilis* populations grown adjacent to *P. protegens* colonies (Powers et al., 2015). This antibiotic seems to act as an interspecific signaling molecule that inhibits bacterial differentiation at subinhibitory concentrations (Powers et al., 2015).

## Conclusion

Here we provide an overview of the phenotypic and molecular responses of plant-beneficial soil bacilli upon sensing signals from other microorganisms that can be encountered in the rhizosphere niche. It is clear that BSMs production by *Bacillus* can be modulated upon interactions with other microbes and that key BSM-driven developmental processes may undergo unsuspected changes. It somehow illustrates the flexibility of these bacteria in re-directing their secondary metabolome to adapt environmental fitness upon sensing the presence of neighboring microorganisms. Nevertheless, the molecular mechanisms integrating the perception of exogenous triggers with a regulatory response leading to enhanced production of BSMs still remain unclear.

A significant boost in BSMs production by soil bacilli has been reported in most cases as an outcome from interactions with plant pathogenic fungi. This is of value in the context of biocontrol of fungal pathogens since direct antagonism is considered as the most powerful mode of action for suppression of plant diseases (Fravel, 2005; Frey-Klett et al., 2011; Köhl et al., 2019). By contrast, direct evidence for an impact of interbacteria interactions on the expression of the secondary metabolome in *Bacillus* is still globally missing. Nevertheless, interaction-mediated variations in colony morphology, motility, biofilm formation, or sporulation illustrate how soil bacilli can protect themselves from antimicrobials emitted by bacterial competitors. Such an impact on those key developmental processes should thus be coupled with significant modulation in the production of specific BSMs underpinning these phenotypes. Depending on the concentration, these BSMs would then act as antimicrobials in interference competition or as signals in cooperative interspecies communication processes not necessarily affecting the growth of the partners (Bleich et al., 2015; Liu et al., 2018). However, this has yet to be thoroughly demonstrated and future examination of developmental controls for BSMs biosynthesis will likely bring light upon the key principles driving environmental fitness of soil bacilli as intrinsically influenced by interspecies competition.

From an ecological viewpoint, further investigations would also help to better understand why soil amendment with selected bacilli, even at high doses, do not durably impact the composition of the rhizosphere microbiome despite their huge arsenal in antimicrobial weapons (Correa et al., 2009; Chowdhury et al., 2013; Kröber et al., 2014; Qiao et al., 2017) and by contrast with some other bacteria and fungi (Buddrus-Schiemann et al., 2010; Chowdhury et al., 2013; Erlacher et al., 2014; Thomas and Sekhar, 2016; Wu et al., 2016). Those bacilli may thus provide protection to their host plant toward microbial pathogen ingress but would avoid detrimental effect on its naturally selected beneficial microbiome which is of prime interest for future application as biocontrol agents.

## Author Contributions

SA, TM, and MO conceived the idea, designed the outlines of the review, and wrote the manuscript. All authors listed have made a substantial, direct and intellectual contribution to the work, and approved it for publication.

## Conflict of Interest

The authors declare that the research was conducted in the absence of any commercial or financial relationships that could be construed as a potential conflict of interest.
